# A novel 5-ring multifocal electroretinography stimulus for detecting hydroxychloroquine retinal toxicity

**DOI:** 10.1007/s10633-021-09858-4

**Published:** 2021-11-11

**Authors:** Adrian Tsang, Pushpinder Kanda, Chloe Gottlieb, Gianni Virgili, Lynca Kantungane, Stuart Coupland

**Affiliations:** 1 Department of Ophthalmology, The University of Ottawa Eye Institute, Ottawa, ON Canada; 2 Eye Clinic, Department of Neuroscience, Psychology, Pharmacology and Child Health (NEUROFARBA), University of Florence, Florence, Italy; 3 Centre for Public Health, Queen’s University of Belfast, Belfast, UK; 4 Ottawa Hospital Research Institute, The Ottawa Hospital, Ottawa, ON Canada

**Keywords:** Hydroxychloroquine retinopathy, Chloroquine retinopathy, Multifocal electroretinogram, Ring ratio

## Abstract

**Purpose:**

Multifocal electroretinogram (mfERG) shows great utility as a screening tool to detect early hydroxychloroquine (HCQ) retinopathy, but its widespread use is limited by the lack of accessibility and long test duration. In this study, we evaluated a novel concentric 5-ring mfERG stimulus to provide a simplified and rapid protocol for screening HCQ toxicity.

**Methods:**

Patients referred for HCQ retinopathy screening were consented to this observational cross-sectional study. Patients with amblyopia, high refractive error (more than 8 diopters), other retinal diseases precluding appropriate evaluation or history of retinal surgery were excluded. The data were collected from patients undergoing HCQ screening at a single center from July 2019 to March 2020. Patients were tested with the new concentric 5-ring mfERG stimulus, standard 61-hexagon mfERG stimulus, spectral domain optical coherence tomography and automated 10-2 visual fields. For the main outcome, the 5-ring mfERG was compared to 61-hexagon stimulus to determine the time-to-test completion and assess the association between ring (R1–R5) amplitude and ring ratio compared against cumulative dose, dose by real body weight and duration of therapy using Pearson correlation.

**Results:**

In total, 52 patients (104 eyes; 5 males and 47 females) were recruited with a mean age of 59 years (range 23–85 years). The 5-ring protocol was markedly quicker to perform (1.3 ± 0.2 min; mean (SD)) compared to the 61-hexagon protocol (5.2 ± 0.6 min), *p* < 0.0001; *n* = 10 patients. The new R2/R5 ring ratio showed a moderate correlation with daily dose (*r* = − 0.640), cumulative dose (*r* = − 0.581) and duration of therapy (*r* = − 0.417). Similar correlations were observed with the new R2/R4 ring ratio which were not significantly different from the new R2/R5 correlation coefficients. The new R2/R5 ring ratio demonstrated a stronger correlation with daily (*p* = 0.002) and cumulative dose (*p* = 0.0001) compared to the 61-hexagon stimulus.

**Conclusions:**

In this exploratory study, our novel 5-ring mfERG protocol significantly shortened data acquisition time while providing comparable results to the standard 61-hexagon stimulus for detecting HCQ-induced electrophysiological changes that are correlated with HCQ dosages and treatment duration. Our protocol has the potential to be more clinically practical by simplifying routine screening.

**Supplementary Information:**

The online version contains supplementary material available at 10.1007/s10633-021-09858-4.

## Introduction

Hydroxychloroquine (HCQ or plaquenil) is a first-line disease-modifying antirheumatic drug (DMARD) used to treat various rheumatological and dermatological diseases including systemic lupus erythematosus and rheumatoid arthritis [1]. Unlike other synthetic and biologic DMARDs, HCQ is more readily available to patient due to its relative lower cost. In addition, studies have shown an emerging role of HCQ in treating neurological diseases, as an adjunct for cancer therapy, and improving hyperglycemic control and lipid profile [1, 2]. Despite the therapeutic benefits, physicians need to carefully consider the risks of HCQ therapy and its more toxic predecessor, chloroquine (CQ or aralen). Retinal toxicity remains a well-known side effect of HCQ/CQ, and the risk to an individual depends on the dose and duration of therapy [3]. The overall prevalence of HCQ retinopathy has been estimated at 7.5% but can exceed 30% and 50% with the dose above 5.0 mg/kg and when used for over 15 and 20 years, respectively [4]. It is not clear if there is any true safe dose with long-term medication use as reports of HCQ retinopathy have been described for cumulative doses as low as 57 g [5]. Risk factors for the development of retinal toxicity include the duration of use > 5 years, excessive daily dose by real body weight (RBW), concurrent tamoxifen use, certain cytochrome P450 gene polymorphisms, and pre-existing retinal, hepatic and renal disease [3].

Most patients who develop early retinal toxicity have minimal visual symptoms (*e.g.,* paracentral scotoma); however, continuous exposure can lead to foveal involvement resulting in vision loss. For unknown mechanisms, HCQ retinopathy can progress despite discontinuing the drug and the severity of structural or functional loss depends on the stage at which the disease was detected [6, 7]. As such, early detection of disease and cessation of HCQ/CQ therapy (particularly before structural retinal pigment epithelium damage) are paramount to limiting disease progression [6]. Unfortunately, the definition of early stage disease prior to which signs of retinal toxicity are reversible remains poorly defined. The current 2016 American Academy of Ophthalmology (AAO) guidelines recommend that a baseline examination of fundus appearance and functional status should be obtained within the first year of initiating therapy using automated visual fields (AVF) and spectral domain optical coherence tomography (SD-OCT) [3]. In the absence of additional risk factors (*e.g.,* macular or renal disease), annual screening can begin after 5 years of exposure with the proper AVF according to race and SD-OCT.

**Fig. 1:**
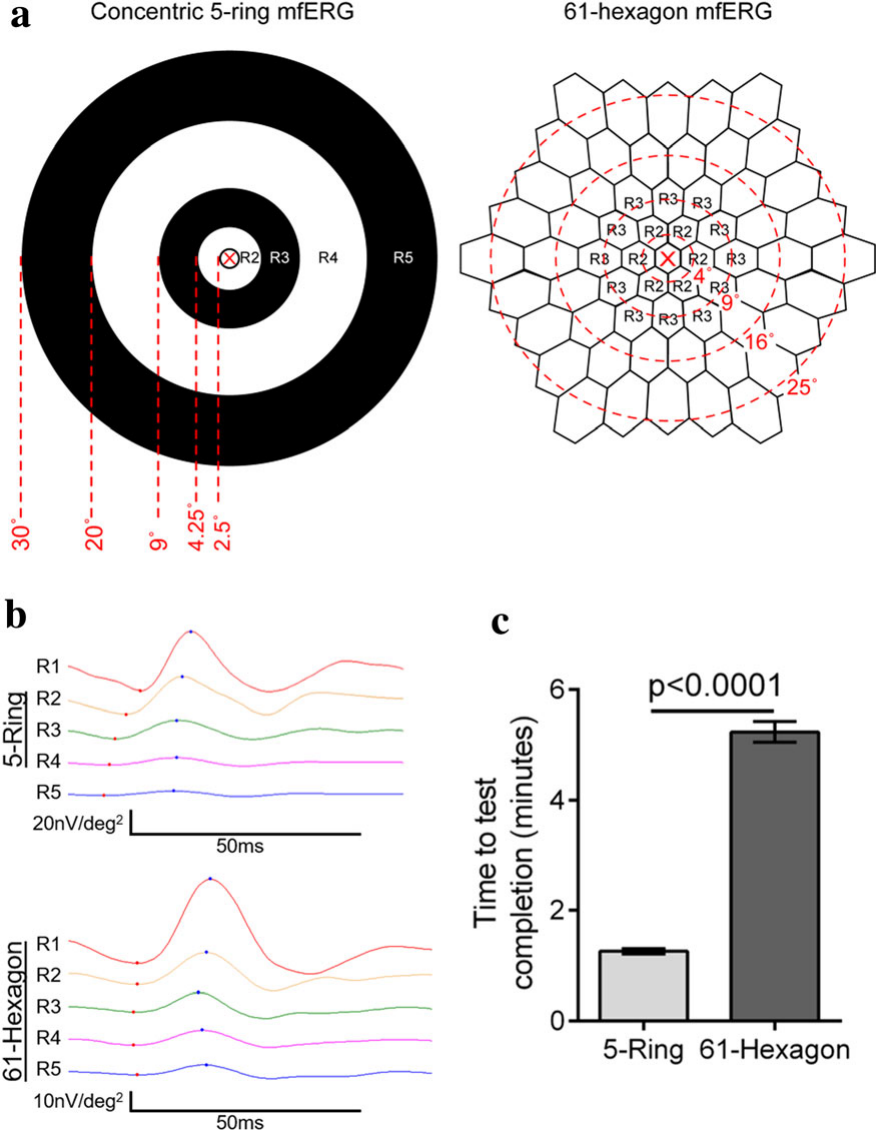
**a** mfERG projection map. The novel 5-ring mfERG stimulus (left) is composed of 5 eccentrically placed rings (R1–R5) compared to the standard 61-hexagon mfERG stimulus (right). The point of fixation (X) is placed in the center. **b** Representative 5-ring and 61-hexagon wave-form plots acquired from the same patient. **c** Graph demonstrating the time taken to complete the 5-ring and the 61-hexagon protocol (mean ± standard error mean, *n* = 10 patients per group)

Although SD-OCT can objectively detect retinal toxicity based on morphological changes, it is not as sensitive compared to functional tests like AVF or mfERG [3, 8]. AVF is readily available for screening, but patient’s subjective response can result in marked variation between tests thereby needing considerable care when interpreting the data. In response to these limitations, mfERG was recommended as an ancillary test to provide objective confirmation of suspected field loss. Despite mfERG having sensitivity akin to AVF, its widespread use is limited by lack of accessibility outside of large clinical centers which is attributable to the need for specialized equipment, and experienced personnel to perform the test and interpret the results. In addition, the standard 61- or 103-hexagonal mfERG can be challenging for the patients as it demands long acquisition time and steady central fixation; testing fatigue can result in the visual gaze drift that produces artifactual signals. In this observational cross-sectional study, we evaluated a novel 5-ring mfERG stimulus that specifically targets the parafoveal region as a screening test for detecting HCQ-related electrophysiologic changes (Fig. 1). We propose that the concentric 5-ring mfERG protocol can simplify and speed up routine HCQ toxicity screening while providing comparable results as the standard 61-hexagonal protocol.

## Methods

This is a cross-sectional study of all the patients referred to the University of Ottawa Eye Institute for HCQ retinopathy screening from July 2019 to March 2020. Data collected on patients included sex, age, best corrected visual acuity (BCVA), refractive status, medications, duration of HCQ (days), body weight, daily dose (mg/Kg) and history of systemic disease including hepatic and renal impairment. Eyes with amblyopia, myopia or hyperopia more than 8 diopters, coexisting retinal disease precluding appropriate evaluation of the retina and prior history of retinal surgery were excluded. Informed consent to have their information collected was obtained from each patient. This cross-sectional study protocol was approved by The Ottawa Health Science Network Research Ethics Board. This study adhered to the tenets of the Declaration of Helsinki.

Each patient underwent a detailed ophthalmic examination with fundus photographs (TRC-50DX; Topcon Medical Systems Inc., Paramus, NJ). Each patient underwent 10-2 AVF (Humphrey Field Analyzer II; Carl Zeiss Meditec Inc, Dublin, CA) and SD-OCT (Spectralis HRA + OCT; Heidelberg Engineering, Heidelberg, Germany) within 1 month of the initial examination. 10-2 AVF was conducted using white SITA testing with pattern deviation plots. AVF results were graded independently by two assessors. A 10-2 AVF with greater than 3 abnormal points (*p* < 2%) within the parafoveal region between 2 and 8 degree from fixation in the pattern deviation plot was considered a positive AVF [9]. Cases with a full or partial ring scotoma or presence of 3 contiguous abnormal points (*p* < 2%) on AVF 10-2 were classified as definite cases of toxicity. SD-OCT central fovea cross-sectional images were reviewed for abnormalities characteristic of toxicity. Disruption of photoreceptor outer segment structural lines (ellipsoid zone line) and thinning of the photoreceptor layers in the foveal and parafoveal regions were classified as evidence of toxicity [3].

Each patient underwent mfERG (Espion Profile Multifocal System; Diagnosys LLC, Lowell, MA) using the standard 61-hexagon mfERG stimulus followed by a novel 5-ring mfERG stimulus at the same visit within 1 month of the initial examination. With the exception of the novel 5-ring mfERG stimulus, all aspects of the mfERGs were performed according to the International Society for Clinical Electrophysiology of Vision procedures. Patients underwent correction of their refractive error prior to testing. Pupil size was measured once at the beginning of testing to ensure appropriate dilation. Patients were dilated (≥ 8 mm) before testing.

A stimulus containing 61-hexagonal elements was projected on the central 30 degrees surrounding the fovea in light adapted subjects’ eyes using an LCD monitor having a static luminance of 400 cd/m2 for ‘on’ segments of the stimuli. Microconductive DTL thread electrodes (Diagnosys LLC, Lowell, MA) were draped on the conjunctiva at the inferior limbus. ERG signals were extracted using an *m*-sequence algorithm (*m*-sequence number of bits equal to 14) in a minimum of eight 30 s epochs.

The novel stimulus consists of 5-ring elements projected on the macula in light adapted subjects’ eyes using an LCD monitor having a static luminance of 400 cd/m2 for ‘on’ segments of the stimuli. The largest ring, Ring 5 (R5) has an outer angle subtense radius of 30 degrees (area of 1570 deg^2). Subsequently, Ring 4 to Ring 1 had a radius of 20 degrees (R4), 9 degrees (R3), 4.25 degrees (R2) and 2.5 degrees (R1), respectively. The parafoveal region of interest in HCQ retinopathy screening is subtended by R2. ERG signals were extracted using an m-sequence algorithm (m-sequence number of bits equal to 12) in a minimum of two 30 s epochs. If the patient had strabismus, each eye was tested separately.

The data were interpreted by S.C. who was blinded to the clinical examination. Individual waveforms composed of the trace arrays were assessed for abnormally like reduced amplitude or prolonged implicit times while ring average analysis was performed using age-matched normative data established at our testing centre. Typical waveforms from both novel 5-ring and 61-hexagon protocols are shown in Fig. 1. The lower limit of test reliability was set at 98% based on industry standard algorithms to assess internal consistency during acquisition time; the algorithm is built into the acquisition software, and mfERG data that failed the reliability index were rejected and the trial was repeated. Trace arrays, ring averages and response density topographic maps were evaluated. Differences of 2 standard deviations or more were classified as abnormal for both 61-hexagon and 5-ring protocols. Ring ratios were computed as a ratio of rings 1 through 4 to ring 5.

The time it took to complete the 5-ring and 61-hexagon protocol was collected randomly from ten additional patients who underwent both testing protocols; the data from these patients were only used to measure time-to-test completion and not used for any other analysis. Based on our previous experience with 61-hexagon protocol and preliminary 5-ring data, we had approximated that *n* = 10 patient per group would be sufficient to show at least a threefold significant difference between groups with an estimated standard deviation of 1–3 min with an alpha error of 5% and power set to 85%. A 2-tailed student’s *t*-test was performed to look for any significant difference between the two groups.

Age distribution was assessed in patients taking less or greater than the daily recommended maximum HCQ dose of 5 mg/kg/day [3]; a 2-tailed student’s t-test was performed to assess for statistical differences. Age distribution was also assessed over a wider daily dosing range by splitting it into quartiles; statistical analysis was performed using one-way ANOVA with Tukey’s post hoc test.

The mfERG parameters were compared between protocols and against cumulative dose, dose by real body weight and duration of HCQ therapy. Ring amplitudes (R1–R5) and ring ratios (R1–R4/R5) were collected for each stimulus protocol. Stata 15.1 software was used to perform the regression analyses and to compute correlation coefficients between variables. Statistical comparisons between Pearson correlation coefficients were performed using the bootstrap method. Graphs were created for visualization using GraphPad Prism version 6.

## Results

In total, 104 eyes (52 patients) were included in the final analysis. There were 5 males and 47 females enrolled with a mean age of 59 years (range 23–85 years; Table 1). Majority of patients had a diagnosis of rheumatoid arthritis (*n* = 24) and systemic lupus erythematous (*n* = 23). The remaining 5 patients were taking HCQ for treatment of Sjögren’s disease, mixed connective tissue disease, granuloma annulare and sarcoid vasculitis. The patients enrolled had been taking HCQ for a mean of 134.5 months (range 2–420 months) with a mean cumulative dose of 1197 g (range 6–4234 g). Twelve patients were taking a daily dose greater than the recommended 5 mg/kg/day for real body weight. There was no statistical difference in the distribution of age amongst patient taking more or less then the recommended daily dose (≤ 5 mg/kg/day) or if the daily dose was split into quartiles (Supplementary Fig. S1). None of the patients included in the analyses had proved toxicity on 10-2 AVF (defined by full or partial ring scotoma or presence of 3 contiguous abnormal points on pattern deviation [*p* < 2%]) or SD-OCT (defined by disruption of photoreceptor ellipsoid zone and parafoveal thinning of the photoreceptor layers). There were no patients that identified as Asian race, and none of the patients were on tamoxifen treatment.

**Table 1:** Patient demographics and characteristics

Patients characteristics (*n* = 52, total 104 eyes)	Value
Sex	
Males (*n* = 5)	9.6%
Females (*n* = 47)	90.4%
Age (years)	
Mean ± SD (years)	59 ± 15
Median (range)	61 (23–85)
Daily HCQ dose per RBW (mg/kg)	
Mean ± SD	4.2 ± 1.4
Median (range)	4.25 (2–7.9)
Cumulative HCQ dose (g)	
Mean ± SD	1197 ± 1019
Median (range)	756 (6–4234)
Duration of HCQ therapy (months)	
Mean ± SD	134.5 ± 106.5
Median (range)	120 (2–420)
Autoimmune disorders	
RA (*n* = 24)	46.2%
SLE (*n* = 23)	44.2%
Sarcoid vasculitis (*n* = 2)	3.8%
Mixed connective tissue disease (*n* = 1)	1.9%
Granuloma annulare (*n* = 1)	1.9%
Sjörgen’s disease (*n* = 1)	1.9%

HCQ hydroxychloroquine; RA rheumatoid arthritis; RBW real body weight; SD standard deviation, SLE systemic lupus erythematosus

The 5-ring protocol was significantly quicker to complete compared the 61-hexagon protocol with the average time-to-test completion of 1.3 ± 0.2 min and 5.2 ± 0.6 min (*n* = 10 patients; *p* < 0.0001), respectively (Fig. 1).

**Fig. 2:**
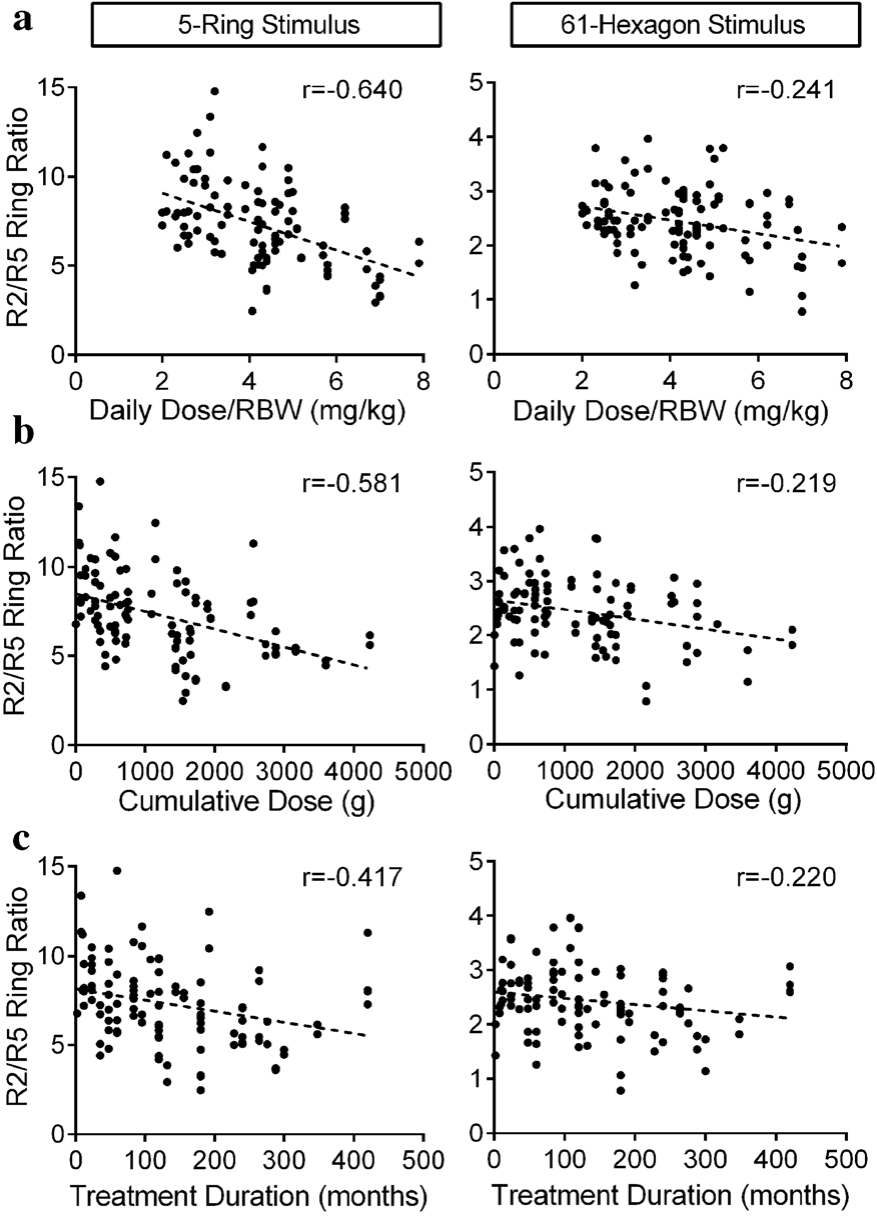
Ring ratio correlation plots. The R2/R5 ring ratio for the novel 5-ring and 61-hexagon stimulus is shown in relation to **a** daily dose adjusted with real body weight (RBW), **b** cumulative dose and **c** duration of therapy. The dashed line represents the linear correlation for each plot

The new R2/R5 ring ratio demonstrated a strong correlation with daily dose (*r* = − 0.640), cumulative dose (*r* = − 0.581) and duration of HCQ therapy (*r* = − 0.417; Fig. 2; all patient data points are shown in Supplementary Fig. S4). This relationship was clearer using the new mfERG protocol rings compared to the 61-hexagon stimulus (Fig. 2). Interestingly, the new R2/R5 ring ratio demonstrated a significantly stronger correlation with HCQ daily (*p* = 0.002) and cumulative dose (*p* = 0.0001) compared to the standard 61-hexagon protocol and *p* = 0.053 for duration of therapy. The new Ring 2 P1 (R2P1) amplitude showed a similar correlation for the duration of therapy (*r* = − 0.418) compared to R2/R5 ring ratio but a weaker correlation with daily (*r* = − 0.328) and cumulative HCQ dose (*r* = − 0.466), Supplementary Fig. S2. Linear regression showed that 32% of the variance in R2/R5 ring ratio (*r*2 = 0.324) and 20% of the variance of the R2P1 amplitude (*r*2 = 0.201) were explained by the cumulative dose of HCQ. Time on HCQ accounted for 20% of the variance of the novel R2/5 ring ratio (*r*2 = 0.205).

The new R2/R4 ring ratio also showed a robust correlation with daily dose (*r* = − 0.595), cumulative dose (*r* = − 0.567) and duration of HCQ therapy (*r* = − 0.444), Supplementary Fig. S3 (all patient data points are shown in Supplementary Fig. S4). This correlation was comparable to the R2/R5 correlation with no statistical different between the two parameters for cumulative dose (*p* = 0.237), daily dose (*p* = 0.098) and duration of therapy (*p* = 0.8). This new ring ratio also showed a stronger correlation with HCQ risk factors compared to the 61-hexagon R2/R4 ring ratio (*p* = 0.007, *p* = 0.0002, *p* = 0.003 for HCQ daily dose, cumulative dose and duration, respectively).

## Discussion

This pilot study was the first application of a concentric 5-ring mfERG stimulus created specifically to isolate the parafoveal region for detecting HCQ retinopathy. Our results demonstrated that the new 5-ring protocol is equivalent to the standard 61-hexagon protocol to detect electrophysiological changes correlated with HCQ risk factors (dose and duration of medication). Importantly, the simplicity of the 5-ring stimulus significantly shorted test completion time which is essential for improving testing efficiency and reliability by minimizing patient fatigue that is responsible for producing artifact signals from movement and loss of target fixation. Currently, the 2016 AAO guidelines have relegated mfERG to an ancillary test due to the lack of accessibility [3]. As such, improving test efficiency by using the concentric 5-ring protocol will bring mfERG one step closer to becoming a routine primary screening tool.

We also demonstrated that the concentric ring design has the potential to be more sensitive to detecting HCQ induced electrophysiologic change in the parafoveal region. The R2/R5 ring ratios and R2P1 amplitude acquired using our new mfERG protocol correlated strongly with HCQ dosing and treatment duration. These correlations were more robust when compared to equivalent parameters of the 61-hexagon stimulus. This stronger correlation may be attributable to the enhanced signal-to-noise ratio produced by the complete coverage of the parafoveal region by a single eccentric ring (Ring 2) compared to the overlap by two eccentric rings (Ring 2 and 3) with 61-hexagon stimulus. In the development of our new ring protocol, the repeatability of R1 (radius of 2.5 degrees) was poor compared to R2–R5, and thus, we recommend not using any smoothening function or averaging of the rings in any future analyses. We also found that the new R2/R4 ring ratio showed a strong correlation with HCQ dose and duration albeit not as strong as the relationship to R2/R5 ring ratio. As such, future studies will explore the new R2/R4 ring ratio as it may provide an additional reliable measure of electrophysiological function in the parafoveal area. This will become particularly important for testing patients requiring a corrective lens (reducing rim artifact) or poorly dilated patients (reducing the logistical burden).

Given the low incidence of HCQ toxicity, our study was restricted to a relatively small number of cases (*n* = 52) at a single tertiary center thereby limited the power of statistical analysis. In spite of this, our study recruited a range of patients with varying levels of exposure to HCQ needed to build strong correlations that were comparable to previously validated 61-hexagon protocol [10]. Future studies will focus on recruiting larger number of patients at multiple centers to validate our results. Another limitation was that during this study period, none of the patients tested had confirmed retinal toxicity using other HCQ screening modalities (AVF, SD-OCT or bull’s eye maculopathy on fundus examination); thus, we could not directly measure the sensitivity of the 5-ring protocol (or the 61-hexagon). In future studies, we plan to compare the concentric 5-ring protocol to other conventional diagnostic modalities (e.g., SD-OCT) in patients with defined HCQ retinopathy. Given that the incidence of abnormal parafoveal electrophysiological signal increased in patient taking higher HCQ dose or longer treatment duration, our results likely represent early detection HCQ retinopathy. These findings are consistent with previous studies which showed that mfERG provides sensitive and subjective measure of early functional changes in patients taking higher cumulative doses [8, 11, 12]. However, further longitudinal studies are needed to confirm if patients with subclinical decrease in mfERG amplitude represent or predict future diseased state and whether or not these patients would benefit from drug cessation. This study was not designed to collect normative data from non-treated patients using the 5-ring protocol (the 61-hexagon did have an institute based normative data) as such effects on data from age-related parafoveal changes were not available. However, age is unlikely to influence our analysis as the distribution of age was nearly equal between patient taking low (≤ 5 mg/kg) and high (> 5 mg/kg) daily dose of HCQ (Supplementary Fig. S1). Lastly, none of the patients enrolled in this study were taking tamoxifen and there were no patients of Asian descent recruited in this study. Future studies will focus on using the new R2 and R3 parameters to characterize potential changes in Asian patients beyond the macula.

Overall, this proof of concept demonstrated that our novel concentric 5-ring mfERG protocol can markedly decrease data acquisition time and simplify routine HCQ toxicity screening compared to the standard 61-hexagon mfERG.

## Supplementary Information

Below is the link to the electronic supplementary material.

Supplementary file1 (DOCX 476 KB)

## Data Availability

The datasets generated and/or analyzed during the current study are available from the corresponding author on reasonable request.
