# Supplementary material for: A novel 5-ring multifocal electroretinography stimulus for detecting hydroxychloroquine retinal toxicity
Source: Doc Ophthalmol. 2021 Nov 11;144(2):117–24. doi: 10.1007/s10633-021-09858-4 (PMC9033700; doi:10.1007/s10633-021-09858-4)
Supplement: Supplementary file 1 — Supplementary file1 (DOCX 476 KB) [file 10633_2021_9858_MOESM1_ESM.docx]

**Supplementary Information**

**Journal:** *Documenta Ophthalmologica*

**Title:** A novel 5-ring multifocal electroretinography stimulus for detecting hydroxychloroquine retinal toxicity

**Authors:** Adrian Tsang, MD^1*^; Pushpinder Kanda, MD/PhD^1*^; Chloe Gottlieb, MD^1^; Gianni Virgili, MD^2,3^; Lynca Kantungane , COMT^1^; Stuart Coupland, PhD^1,4^

*Indicates co-first authorship

**Affiliations:**

1. The University of Ottawa Eye Institute, Department of Ophthalmology, Ottawa, Ontario, Canada.
2. Eye Clinic, Department of Neuroscience, Psychology, Pharmacology and Child Health (NEUROFARBA), University of Florence, Florence, Italy
3. Centre for Public Health, Queen’s University of Belfast, United Kingdom
4. Ottawa Hospital Research Institute, The Ottawa Hospital, Ottawa, Canada

**Correspondence to:**

Dr. Pushpinder Kanda

University of Ottawa Eye Institute

The Ottawa Hospital, General Campus

501 Smyth Rd. Ottawa, Ontario, Canada K1H 8L6

Email: pkand042@uottawa.ca

Phone: (613) 798-5555

Fax: (613) 739-6635

**

**

**Supplementary Fig. S1** Age distriubtion. Box and whisker’s plot on the left shows the distribution of age in patients taking less or greater than the recommended maximum daily HCQ dose (5mg/kg per day). The plot on the right shows the age distribution among daily doses split into quartiles (1^st^ quartile 2.0-3.1, 2^nd^ quartile to 4.3, 3^rd^ quartile to 5 and 4^th^ quartile to 7.9 mg/kg)

**

**

**Supplementary Fig. S2** Ring 2 P1 amplitute correlation plot. The novel 5-ring R2P1 amplitude correlation plot for daily dose adjusted with real body weight (RBW), cumulative dose, and duration of therapy. The dashed line represents the linear correlation for each plot

**

Supplementary Fig. S3** R2/R4 ring ratio correlation plot. The R2/R4 ring ratio for the novel 5-ring and 61-hexagon stimulus are shown in relation to (**a**) daily dose adjusted with real body weight (RBW), (**b**) cumulative dose, and (**c**) duration of therapy. The dashed line represents the linear correlation for each plot

**

**

**Supplementary Fig. S4** Ring ratios for 5-ring and 61 hexagon protocol. Strip chart showing all data point for R2/R5, R2/R4, and R2/R3 for 5-ring and 61-hexagon protocol; each colour & shape corresponds to the same eye between the two protocols. The data points were divided into two time point, patients on HCQ for <120 months and ≥120 months.
